# Ultrafast switching of optical properties in a doped semiconductor by intense femtosecond laser pulses

**DOI:** 10.1038/s41377-026-02346-x

**Published:** 2026-08-04

**Authors:** Yan Li, Stefano Vezzoli, Tim Klee, Joseph J. Broughton, Romain Tirole, Anthony C. Harwood, Hortense Allegre, Simon A. R. Horsley, Riccardo Sapienza, John B. Pendry, John W. G. Tisch

**Affiliations:** 1 The Blackett Laboratory, Department of Physics, Imperial College London, Exhibition Rd, London, SW7 2BW UK; 2 Department of Physics and Astronomy, Centre for Metamaterial Research and Innovation, University of Exeter, Stocker Road, Exeter, EX4 4QL UK

**Keywords:** Ultrafast photonics, Nonlinear optics

## Abstract

The rapid switching of materials when excited by ultrashort pulses of light is central for many optical technologies, and in particular to the developing field of time-varying metamaterials. These out-of-equilibrium interactions are difficult to capture with traditional theoretical models. Here we combine experiments and theory to unravel different regimes of interactions and a response saturation for a 44 fs, near-infrared pump pulse exciting a switchable doped semiconductor indium tin oxide thin film target. We model this process as a change in plasma frequency due to the excitation of hot electrons in a non-parabolic conduction band, which increases their effective mass. Our calculations show that saturation at high pump intensities arises because the pump heavily depopulates electrons from below the Fermi level. Excellent agreement with values extracted from experimental data confirms our model. For lower pump intensities, a two-temperature model is consistent with our data, but at higher intensities, it is apparent that other processes are at work, which we attribute to Auger transitions from the valence band, which introduce complex structure into the response, due to non-equilibrium rearrangement of energy between electrons and holes.

## Introduction

Intense ultrashort laser pulses change the electronic state of a solid on femtosecond timescales, which in turn rapidly changes the response to interactions with a probe laser pulse. Many applications require rapid, efficient switching, such as signal processing, optical computing, and the fast-developing field of time-varying metamaterials. Epsilon-near-zero (ENZ) materials have recently attracted considerable interest due to both their linear and nonlinear optical properties^1–4^. Films of transparent conductive oxides like indium tin oxide (ITO) and aluminium zinc oxide, which are highly conductive yet transparent in the visible spectrum, display relatively low losses in the near-infrared, high third-order nonlinearities, and large damage thresholds^5,6^. Their non-perturbative nonlinear response^7^ has been exploited for third harmonic generation^8,9^, high-order harmonic generation^10–12^, optical modulators^13–15^, and demonstrations of time-varying effects at optical frequencies^16–18^.

In particular, using laser pulses to rapidly switch ITO’s optical transparency has become a key method to investigate ultrafast time-varying physics in the optical domain^5,12,19–21^. At relatively low pump intensities, the two-temperature model^5,16,22–24^ fits post-pump shifts in probe reflectivity and transmissivity, and their recovery time. It also fits red shifts in reflection spectra within the time resolution of experiment. Previous work^17^ deploying ‘slits in time’ revealed excitation processes of the order of an optical cycle, which are yet to be fully explained. At high pump intensity, saturation of both modulation amplitude and spectral shift occurs, and experiments suggest complex relaxation processes, some on short time scales^25^. Achieving excitation and relaxation times on the order of the optical period (~ few fs) could enable applications such as photonic time crystals^26–28^ or advanced light synthesis^29,30^.

Here we present pump-probe experiments supported by theory that reveal the dynamics of this modulation process. While we focus on ITO, our conclusions have wider relevance to ultrafast optical switching in transparent conducting oxides^31^. We study the time- and frequency-resolved response of a 310 nm thick ITO film, pumped by 795 nm pulses, up to 12.7 TW/cm^2^, and probed by a weak pulse at 1240 nm, close to the ITO ENZ wavelength. In both transmission and reflection, we observe saturation above 3 TW/cm^2^, along with large spectral shifts. From the ITO band structure, we model the pump-induced changes in the effective mass of electrons that explain the plasma frequency shift. Saturation is attributed to heavy depopulation of the electrons below the Fermi level. Our calculations of the plasma frequency shifts and saturation intensities agree well with experiment. We also explain the shortening of the switching time at saturation: once sufficient energy has been absorbed, switching can occur within a few femtoseconds — faster than the pump duration. At intensities above 9 TW/cm^2^, reflection and transmission partially recover, indicating a new, fast, non-equilibrium process. We attribute this to excitation of the valence band electrons by sequential absorption of several photons, followed by an Auger process.

## Model

**Fig. 1:**
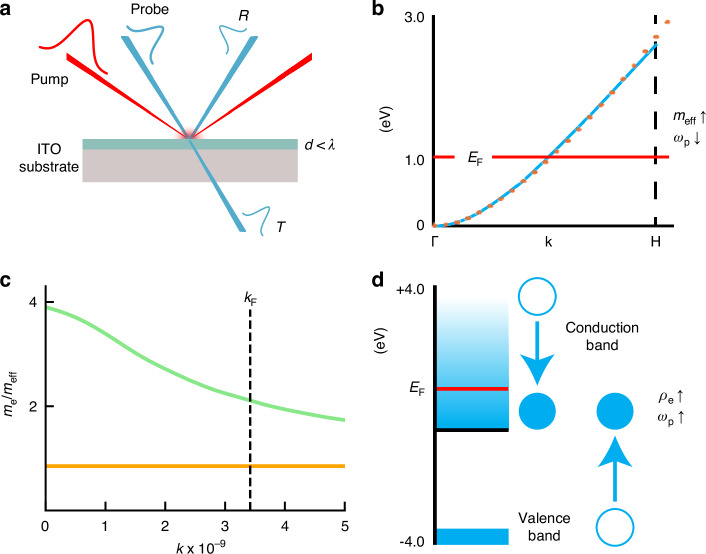
Dynamics under intense and short laser pulses. **a** Schematic of the pump-probe experiment in a doped semiconductor thin film of ITO. A short pump pulse creates a large modulation of Fresnel coefficients in time, which is probed by a weaker pulse. **b** The Kane fit (red dots) to the ITO band structure along *Γ* − H (cyan line), showing the Fermi energy before excitation (red line). The pump induces intraband electronic transitions in a non-parabolic conduction band, leading to an increase in effective mass and hence a decrease in plasma frequency. **c** Inverse effective mass of electrons, normalised to the inverse free electron mass (green curve), as a function of wavevector, showing most of the changes happen at and below the Fermi wave vector given in units of ${{\rm{m}}}_{e}^{-1}$. The orange curve gives the asymptotic value. **d** Schematic occupancy of energy levels after excitation indicated by blue shading, now showing the valence bands 3.9 eV below the bottom of the conduction band. Further increasing the pump intensity beyond saturation gives more than one photon to each electron on average. Absorption of three photons or more is enough to initiate Auger processes whereby an energetic electron gives some of its energy to a valence electron, knocking it into the conduction band, and leaving a hole in the valence band

A short pump pulse focused onto a thin material film can induce large relative changes of the permittivity which result in near-unity changes of the complex refractive index around the ENZ condition, producing time-varying Fresnel coefficients experienced by a weak probe pulse (Fig. 1a). For conducting materials such as ITO, this ultrafast change in optical properties can be described by modulation of the plasma frequency, *ω*_p_, in the Drude model. We model the evolution of *ω*_p_ over short timescales as a function of deposited pump energy by tracking electron dynamics in the conduction band.

At low pump intensities, the electron response has three stages: (i) initial pump energy absorption and promotion of conduction electrons to higher energies as “hot electrons” in the same band; (ii) thermalisation of the hot-electron population through electron-electron interaction; and (iii) slower equilibration through interaction with lattice phonons, well-explained by the two-temperature model^5^. The first two stages occur more rapidly than the pump or probe duration and are less studied. In this regime, excitation of conduction electrons to higher energy increases their effective mass, suppressing *ω*_p_, leading macroscopically to induced transparency.

Our theory begins with the conduction band dispersion *E*(*k*) ^32,33^, fit using the non-parabolic Kane model^34^. The fitting parameters are given by,

1 $$\begin{array}{rcl}E(k) & = & {a}_{{\rm{K}}}(-2{b}_{{\rm{K}}}+{k}^{2}/{G}^{2}+2\sqrt{{k}^{2}/{G}^{2}+{b}_{{\rm{K}}}^{2}})\\ {a}_{{\rm{K}}} & = & 1.15\,\mathrm{eV},\,{b}_{{\rm{K}}}=0.26\,(\mathrm{dimensionless}),G=6.22\times 1{0}^{9}{{\rm{m}}}^{-1}\end{array}$$

The fitting is shown in Fig. 1b where the dots of the Kane model are almost hidden by the full line of the band structure calculations. From *E*(*k*) we calculate the effective mass as a function of wave vector *k*, ${m}_{{\rm{eff}}}^{-1}(k)={\hslash }^{-2}{k}^{-1}dE/dk$, shown in Fig. 1c. For a non-parabolic band, we show later in Methods that this is the correct definition rather than ${m}_{{\rm{eff}}}^{-1}(k)={\hslash }^{-2}{d}^{2}E/d{k}^{2}$. The two differ substantially for ITO. Using ${\omega }_{{\rm{p}}}^{2}={\rho }_{e}{e}^{2}{m}_{{\rm{eff}}}^{-1}({k}_{{\rm{F}}}){\varepsilon }_{0}^{-1}$ and ${\rho }_{e}={k}_{{\rm{F}}}^{3}/3{\pi }^{2}$, where *ρ*_*e*_ is the electron density and *k*_F_ is the Fermi wavevector, we calculate *ρ*_*e*_, *k*_F_, and ${m}_{{\rm{eff}}}^{-1}({k}_{{\rm{F}}})$ from the plasma frequency, *ℏω*_p_ = 1.97 eV, to obtain *ρ*_*e*_ = 1.35 × 10^27^ m^−3^, *k*_F_ = 3.42 × 10^9^ m^−1^, *m*_*e*_/*m*_eff_(*k*_F_) = 2.06, and *E*_F_(*k*_F_) = 1.15 eV. ITO is a degenerate semiconductor, with the Fermi level inside the conduction band due to Sn doping. Its abundance of free electrons allows it to withstand pump intensities of several TW/cm^2^.

Before pumping, we assume a cold electron gas, *T* = 0, so only *m*_eff_(*k*_F_) contributes, since *n*_F_ is constant everywhere except for a step at the Fermi surface. As conduction electrons absorb photons from the pump, the occupancy of the conduction band *n*(*E*) changes, altering the effective mass (as illustrated in Fig. 1b and c), due to the non-parabolic nature of the conduction band. Electrons are assumed to thermalise amongst themselves immediately after passage of the pump, but not with the lattice^35^. Our calculations are consistent with the commonly assumed increase of mass and shift of *ω*_p_ to lower frequencies. Saturation of *ω*_p_ is predicted when nearly all the electrons have been promoted from below the Fermi level, due to removal of electrons from below the Fermi surface, where variation in mass is most significant, to states with high *k* but only small variations of the effective mass due to its asymptotic behaviour (see Fig. 1c). Consequently, for high pump intensities, saturation occurs earlier in the pumping process, further input of energy having little impact on reflectivity and transmissivity. From the electron density, we can estimate the saturation intensity. If each electron absorbs one photon, this requires an input energy of 8.43 × 10^−3^ J/cm^2^. Saturation only becomes significant around 3 TW/cm^2^ because many electrons absorb more than one photon. Although the asymptotic value of *m*_eff_ is not well known due to the complexity of the band structure, at large *k* well above *k*_F_, its value can be fitted from pump-probe data.

Once most electrons have been promoted above the Fermi energy around 3 TW/cm^2^, the chemical potential becomes negative, and conduction electrons reach extremely high temperatures. For the highest pump intensities, absorption of multiple photons produces many electrons above the bandgap. We propose that this initiates Auger processes, adding conduction electrons and creating holes in the valence band, as illustrated in Fig. 1c. Auger processes happen through exchange of a virtual photon between two electrons: one falls down in energy, the other rises. It is the dominant mechanism for thermal equilibrium amongst electrons in the conduction band.

**Fig. 2:**
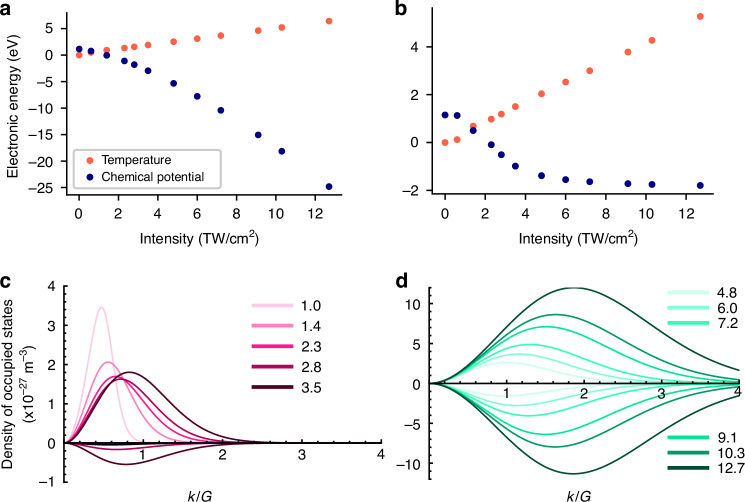
Electron temperature and hole excitation at extremely high intensities. Top row: calculated chemical potential and electron temperatures in ITO in eV, given the conduction electron density and pump intensity input as described in the text. **a** On the left, assuming no excitation of the valence band. **b** On the right, allowing valence band excitation and assuming a simple band structure that is the mirror image of the conduction band. Bottom row: calculation of the density of occupied states as a function of wavevector in units of *G*, the reciprocal lattice vector. Negative numbers indicate hole states in the valence band. **c** Calculated density of occupied states for electrons (positive values on y-axis) and holes (negative values), for a sequence of lower pump intensities in TW/cm^2^. **d** A sequence for higher pump intensities. The data points are for the same intensities used in (**a**) and (**b**). The threshold wavevector for Auger processes is *k*/*G* = 1.2, corresponding to an energy of 3.9 eV

Figure 2 shows two scenarios, and provides an approximate theory that indicates underlying processes with the valence- and conduction- band population. The first assumes thermal equilibrium only amongst the conduction electrons (Fig. 2a) and ignores the possibility of exciting valence electrons. Given the large amounts of energy absorbed from the pump, the rise in electron energy is inevitable and more than enough to share their energy with valence electrons, which forces us to examine that possibility. This we do using a minimalist model where valence bands are represented as an inversion of the conduction band, i.e. we consider only the nearly-free-electron component of these bands. Consideration of the very high density of tight-binding valence bands would only strengthen our argument for excitation but would add complexity beyond the scope of this paper. We assume in Fig. 2b that the valence bands are part of the thermal equilibrium, considerably increasing the population of electrons in the conduction band. We justify this assumption by recognising that the e-e interaction required for valence band excitation is generally a much faster process than the e-phonon processes which remove heat. Certainly, e-e processes acting within the conduction band are widely assumed in the literature to operate on a timescale faster even than the pump width. We make the point that valence band excitation cannot be neglected at high pump powers. Current theories do not explain the data; further work will be required to confirm the Auger model presented here. This increases *ρ*_*e*_, and hence *ω*_p_, acting in opposition to mass increase, and thus further contributing to the saturation of *ω*_p_ and even its overall rise at extremely high pump intensities (as sketched in Fig. 1d). After the pump, electrons cool via phonons, which control the electron-hole recombination rate. The electron density decays until almost all the holes are filled and the original conduction electron density is restored. Subsequent cooling conserves the number of conduction electrons. Hence, heating and cooling dynamics in the high-intensity regime are more complex than at lower intensities. Given an absorption coefficient calculated and measured to be 15%, the result is a temperature rise of 6.43 eV according to Fig. 2a or 5.27 eV according to Fig. 2b. The temperature rise is almost linear in both cases for input powers above about 1.5 TW/cm^2^. Although approximate and thus presumably overestimating the conduction electron temperature, this model serves to illustrate the likely equilibration mechanism between the conduction and valence bands after the absorption of the pump pulse. As a result, we calculate that in both cases the specific heat is asymptotic, taking a value of about 4.0 × 10^4^ Jm^−3^K^−1^. We later use a more accurate temperature-dependent heat capacity^35^ to estimate the parameters of ITO from fitting to a two-temperature model (See Methods). This is much higher than at low temperatures, where only electrons within *k*_B_*T* of the Fermi energy participate.

Once most of the electrons are excited, the specific heat rises. This is evidenced by a kink in Fig. 2b in the temperature plots at around 1.5 TW/cm^2^. Figure 2c gives results for pump intensities up to 3.5 TW/cm^2^ and shows little hole creation. Here the two-temperature model fits well. In contrast, Fig. 2d shows a dramatic increase in hole creation. Many additional bands beyond our simple Kane two-band model are likely involved. This resembles avalanche diode breakdown, where strong electric fields accelerate conduction electrons sufficiently to initiate hole creation. We argue that hole creation cannot be neglected at higher pump intensities.

## Results

**Fig. 3:**
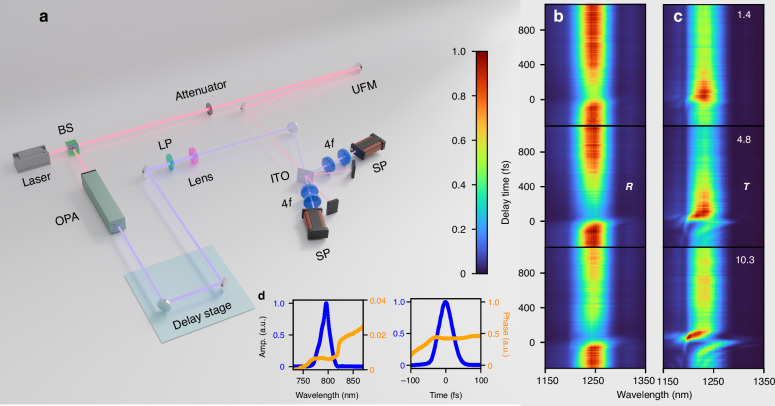
Schematic of the pump-probe experiment to track the ultrafast variations in ITO at extremely high pump intensities and an example of raw data. **a** The experimental setup uses a 795 nm, 44 fs pump laser pulse and a 1240 nm, 50 fs probe laser pulse. 4f imaging is used to ensure large-angle collection, due to potential spatio-temporally induced beam deflection and fission of the probe^18^. Collected signal is analysed by calibrated spectrometers. OPA: optical parametric amplifier. BS: beam splitter. UFM: ultrafast-compatible focusing mirror. SP: spectrometer. LP: long pass filter. 4f: 4f recollimation and re-collection line. **b**, **c** The reflected and transmitted spectra as a function of the pump-probe delay, for a selection of the low (1.4 TW/cm^2^), medium (4.8 TW/cm^2^), and high (10.3 TW/cm^2^) pump intensities. **d** The temporal and the spectral profile of the pump pulses. The flat phases across the pulse envelope show they are nearly dispersion-free

The theoretical model described so far can be compared to experiments using time-resolved pump-probe spectroscopy. Measurements were performed on a 310 nm ITO film deposited on a 1.1 mm float glass substrate. The sample was excited with 795 nm, 44 fs near-infrared pump pulses (Fig. 3a), and probed with 1240 nm, 50 fs pulses. The delay between pump and probe was scanned over a range of 3 ps to record the optical modulation. The ENZ wavelength of the 310 nm ITO film – where the real part of its permittivity crosses zero according to the Drude model – coincides with the 1240 nm probe. The intensity of the probe pulses was kept below ~ 10 GW/cm^2^ to avoid unwanted nonlinear effects. The pump photon energy *ℏω*_pump_ = 1.56 eV is greater than the Fermi energy. The ENZ resonance at *ℏω*_0_ = 1 eV is far below *ℏω*_pump_ and in our experiment plays no direct role in absorption of pump radiation. Pump intensities were extended to the strong field regime (several TW/cm^2^) to investigate the intensity dependence of the nonlinear effect in ITO. Both beams in p-polarisation were focused on the sample with oblique incidence angles (pump ~ 50^∘^ and probe ~ 45^∘^, ± 5^∘^). The off-normal angular incidence was selected for optimal modulation per unit shift of the plasma frequency^24^. The probe wavelength was chosen to couple to the Berreman mode^36,37^, in order to enhance any change of the refractive index induced by the pump, thus enabling effective resolution of the dynamics^38^. The probe pulses were then recollimated and re-focused through a 4f lens system to ensure the probe signal was collected, even after strong modulations that can cause recently reported spatio-temporal diffraction and fission^18,30^. The probe signal was then collected through optical fibres and sent to a spectrometer. Varying the pump-probe delay enabled detailed investigation of time-dependent nonlinear spectral and amplitude modulations, as illustrated in Fig. 3b for reflection, *R*, and Fig. 3c for transmission, *T*.

A significant change in reflectivity and transmissivity occurs at the air-ITO interface under optical excitation. We divide the range of pump intensities into three regimes: “low" (0 to 3 TW/cm^2^), “medium" (3 to 9 TW/cm^2^), and “high" intensity (9 to 12.7 TW/cm^2^). Each colour map shows a representative intensity in each range. First, we observe abrupt amplitude modulation around *t*_0_. The rapid drop in reflectivity produces a dip, while the corresponding rise in transmission leads to a peak in the spectrogram, as shown in Fig. 3b and c. Moreover, spectral redshifts appear at negative delays (*t* < *t*_0_) and blueshifts at positive delays (*t* > *t*_0_), respectively.

These are due to ultrafast and large changes in the phase of the Fresnel coefficients, and are also observed in the glass substrate alone, consistent with cross-phase modulation (XPM) in the substrate (see SI). In transmission, the spectral shifts due to the “slow”^39^ modulation of the Fresnel coefficients in ITO cannot be fully separated from the contribution of XPM in the substrate, as well as of XPM in the ITO itself. Hence, we will focus our analysis and modelling on the spectrally integrated amplitude modulation, although the magnitude of spectral shifts will be used to bound the fitting parameters. As observed on the substrate alone, XPM mainly leads to a redistribution of energy at different frequencies, but does not change the amplitude significantly, and cannot explain the very large modulations of *R* and *T* that we observe in ITO. To isolate the ITO dynamics, we analyse the reflected probe, which does not propagate into the substrate. Moreover, any spectral shift recorded at *t* > + 50 fs will not be affected by “fast”, i.e., parametric nonlinearities, as there is no temporal overlap between pump and probe pulses.

**Fig. 4:**
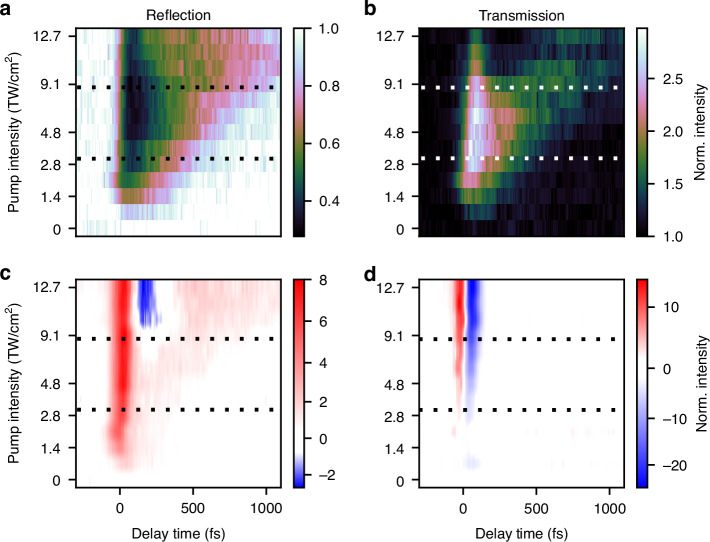
Saturation of the plasma frequency, and complex dynamics at very high pump intensities. **a**, **b** Reflection and transmission plots of the spectrally integrated probe intensity as a function of the pump-probe delay (*x*-axis) and pump intensity (*y*-axis). The two plots show a nearly symmetric behaviour: an early saturation of the maximum modulation around 3 TW/cm^2^, an initial increase of the relaxation time, followed by an ultrashort decay mechanism for very high intensities and a partial recovery, before the “slow” thermal relaxation. This new decay time is accompanied by significant reduction of the maximum modulation and a breakdown of the standard two-temperature model, suggesting the onset of a new process which counterbalances the increase of effective mass/decrease of *ω*_p_, while introducing a new timescale, which we assign to Auger excitations of valence holes in our model. **c**, **d** Measured spectral shifts of the reflected and transmitted probe. The shifts for transmission around *t*_0_ are strongly affected by nonlinear parametric effects like XPM in the substrate and ITO. However, the emergence of blue shift in *R* at very high pump intensities between 100 and 200 fs, as well as for *T* after 100 fs, is evidence of a new, ultrafast relaxation process

The dynamics of the spectrally integrated signal are summarised in Fig. 4a for reflection and Fig. 4b for transmission. The two colour-plots show a nearly symmetric behaviour. The “peak” in the modulation of transmission corresponds to the “dip" of reflection. This agreement indicates energy transfer between the Fresnel reflected and transmitted probe due to the change in refractive index. A saturation of the maximum modulation occurs around 3 TW/cm^2^, as predicted by the band model, and is accompanied by an initial increase of the thermal relaxation time. This is followed by the emergence of a faster relaxation process at very high intensities and a partial recovery between 100 and 300 fs, before the much slower equilibration with the phonons. Both the reflection and the transmission “fast relaxation feature", appearing shortly after the pump has left the medium (around 100 fs) and before the thermal relaxation process, suggest the emergence of a new dynamical process with a separate relaxation timescale. This new decay time is accompanied by a significant reduction in the maximum modulation and a blue shift in both *R* and *T* spectra (Fig. 4c and d), indicating the breakdown of the two-temperature model and the onset of a new process which counterbalances the decrease of *ω*_p_. Just as a rapid rise in reflectivity produces a red shift, a rapid fall will result in a blue shift, and this is seen in Fig. 4. The blue shift is another strong indication that other processes are at work, not previously identified. The fast relaxation feature and blue shift continue to grow as the incident pump intensity increases. A maximum blueshift of 2.7 nm is recorded at a pump intensity of 12.7 TW/cm^2^ in reflection. As discussed in our theoretical model, we suggest this process relates to the Auger hole-creation and conduction band recombination initiated via strong-field pumping.

## Discussion

**Fig. 5:**
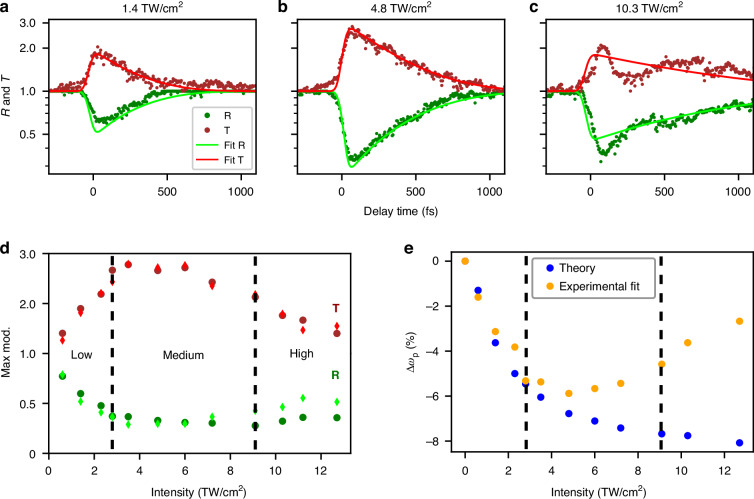
Analysis and discussion of the results. **a**–**c** The spectrally integrated probe signal as a function of pump-probe delay for a selection of pump intensities. Reflection (green) and transmission (red) data points are fitted with a two-temperature model with *ω*_p_ and the time constants as the main fitting parameters. To calculate the time-varying reflection and transmission coefficients from the change in plasma frequency, in the presence of medium dispersion, an operator theory is used^40^. The fit breaks down as the two-temperature model cannot capture the new Auger process in the high-intensity regime. **d** Evolution of the maximum modulation ratio for reflection ${R}_{{\rm{mod}}}/{R}_{0}$ (green dots) and transmission ${T}_{{\rm{mod}}}/{T}_{0}$ (red dots) from the experiment, compared with extracted fit values (green and red diamonds). **e** Values of shift in *ω*_p_ extracted from the fits predicted from the band structure theory, in comparison with the fitted experimental values. The agreement is excellent up to moderate intensities of ~ 3 TW/cm^2^, where the dispersion *E*(*k*) is well known

The amplitude modulation of the reflectivity and transmissivity is shown in Fig. 5a–c together with theoretical calculations following a two-temperature model (see Methods). We observe a rapid rise in reflectivity within ~ 80 fs when the pump arrives. Since the pump photon energy is below the bandgap of ITO, carrier heating occurs via free-carrier absorption. Electrons gain energy and occupy higher-energy states above the Fermi level within the non-parabolic conduction band, resulting in an increased effective mass. This leads to a redshift of the plasma frequency, causing the observed reduction in reflectivity. Thermal relaxation follows the traditional two-temperature model at low pump intensities. At high pump intensities, we expect considerable non-equilibrium rearrangement of energy between valence and conduction electrons on a short time scale, resulting in fluctuations in *ω*_p_. However, the electron distribution once in equilibrium can only be cooled by emission of phonons.

In order to extract information about the electron dynamics and relate the data to our theoretical model, we use the operator theory described in Ref. ^40^, which uses the plasma frequency as the main parameter to calculate the resulting change in the ITO film Fresnel coefficients. The model assumes Gaussian pump and probe pulses with fixed FWHM, but variable pump intensity. As described in the Methods, a full set of parameters (the pump power, pump arrival time, carrier density, electron-phonon coupling, probe wavelength and electron collision rate) was fit to all the spectrally resolved pump-probe data (transmissivity, reflectivity, and frequency shifts due to the modulation). As expected, the probe wavelength, pump arrival time, and carrier density were found to be consistent across all experiments. The model gives good agreement at lower intensities, but above 3—4 TW/cm^2^, the quality of agreement decreases, especially for the spectral shifts. We identify Auger-induced hole creation in the valence band as the likely cause of the breakdown of the two-temperature model.

In Fig. 5d, we summarise our experimental findings on the evolution of the maximum modulation ratio with pump intensity. In the low-intensity regime, the amplitude modulation scales with the pump intensity in both reflection and transmission channels, giving rise to a maximum 280% increase in the transmitted intensity and 74% reduction in the reflected intensity before saturation. Saturation occurs around 3 TW/cm^2^, in good agreement with the theoretical prediction, when each electron in the conduction band has absorbed at least one pump photon. For intensities above ~ 3 TW/cm^2^, both modulations begin to decrease from their peak values.

From the operator theory, we can extract the best value of the plasma frequency for each pump intensity in Fig. 5e. Our theory accurately predicts the evolution of the plasma frequency, as extracted from the fits, by using the value of the effective mass at large wave vectors as the only free parameter. The evolution of both *R* and *T* at high intensities above ~ 3 TW/cm^2^ is not captured by the simple saturation of the effective mass and requires the introduction of a new process, which acts in the opposite direction on the plasma frequency.

Referring to Fig. 2a and b, we note that for an input intensity of around 12 TW/cm^2^, electron temperatures in excess of 6 eV are predicted. This is sufficient energy to initiate Auger processes where electrons are excited out of the valence band, creating a hole, and adding an electron to the conduction band. This changes the charge density and will affect the plasma frequency. More detailed calculations would be complex and were beyond the scope of this work. However, Auger excitation and subsequent decay – being an electron-electron interaction – are expected to proceed much faster than electron-phonon equilibration. We therefore suggest that the fast relaxation feature and blue shift result from the non-equilibrium rearrangement of energy between electrons and holes.

In conclusion, our experiments explore a new energy range of excitation of ITO, now up to an intensity of 12.7 TW/cm^2^. The complex dynamics we observe point to the possibility of a fast decay of modulation, faster than the slow ~ 300-500 fs relaxation time; this has an impact on the realisation of complex temporal phenomena, for example, time-crystals, which require large, rapid modulations of the refractive index. On the theoretical side, we have coupled the two-temperature model to an exact scattering calculation of the probe interacting with a time-modulated medium. However, our experiments reveal the need for revision of current theory when a high pump intensity heats electrons to more than 3 or 4 eV. More complex processes are at work than contained in the two-temperature model. This points to the need to go beyond using, e.g., the equilibrium Fermi-Dirac distribution to model the electron distributions in the conduction band. The model we used is especially suited for transparent conductive oxides and therefore we expect it can be applied to aluminium zinc oxide, other heavily doped semiconductors and potentially other ENZ materials. Exploiting previously calculated electron energy dispersion, *E*(*k*), enables several quantities to be estimated, such as effective mass and electron temperature, given the amount of energy deposited. In this way, we show that electrons in the valence band are almost certainly promoted to the conduction band. Indeed, they must be if thermal equilibrium is established amongst the electrons. Although we have answered some important questions, more challenges remain in this high-intensity regime.

## Methods

### Calculation of the effective mass

A probe is tuned to the resonance at epsilon-near-zero frequency of ITO, *ω*_0_, which marks the boundary between transmitting and reflecting regimes. *ω*_0_ depends on the plasma frequency, *ω*_p_. Before the pump pulse arrives, the sample is assumed to be cold, *T* ≈ 0, with all states below the Fermi level occupied. The inverse effective mass at the Fermi level, 1/*m*_eff_(*k*_F_), determines *ω*_p_. In Eq. (2), we adapt the conventional derivation of the plasma frequency to the case of variable mass when the temperature is finite. We take an average over the Fermi distribution, *n*_F_,

2 $$\begin{array}{rcl}{\hslash }^{2}{\omega }_{{\rm{p}}}^{2} & = & \frac{{e}^{2}}{4{\epsilon }_{0}{\pi }^{3}}{\int }_{0}^{\infty }-\frac{\partial {n}_{{\rm{F}}}}{\partial {k}_{z}}\frac{\partial E}{\partial {k}_{z}}\,{d}^{3}k=\frac{{e}^{2}}{4{\epsilon }_{0}{\pi }^{3}}{\int }_{0}^{\infty }-\frac{\partial {n}_{{\rm{F}}}}{\partial k}\frac{\partial E}{\partial k}{\cos }^{2}\theta \,\,{d}^{3}k\\ {n}_{{\rm{F}}}(k) & = & {({{\rm{e}}}^{(E(k)-{E}_{{\rm{F}}})/{k}_{{\rm{B}}}T}+1)}^{-1}\end{array}$$

We can re-express Eq. (2) in terms of an effective mass (the “transport effective mass” as described in e.g. Ref. ^41^)

3 $${\hslash }^{2}{\omega }_{{\rm{p}}}^{2}=\frac{{e}^{2}}{4{\epsilon }_{0}{\pi }^{3}}{\int }_{0}^{\infty }-\frac{\partial {n}_{{\rm{F}}}}{\partial k}\frac{{\hslash }^{2}k}{{m}_{{\rm{eff}}}}{\cos }^{2}\theta \,{d}^{3}k$$

4 $$\frac{1}{{m}_{{\rm{eff}}}(k)}=\frac{1}{{\hslash }^{2}k}\frac{\partial E(k)}{\partial k}$$

There are several published calculations of *E*(*k*) ^32,33^. Here we use the Medvedeva data for dispersion in the *Γ* − H direction and fit the Kane model^34^. The latter hybridises two bands originating at ± H, intersecting at *Γ* to form a gap, the upper part of which constitutes the conduction band illustrated in Fig. 1.

Since we assume *T* = 0 before pumping, only the value of *m*_eff_(*k*_F_) contributes to Eq. (3) as *n*_F_ is constant everywhere except for a step at the Fermi surface. Using *E*(*k*) we can calculate the density of electrons in the ITO film to be *ρ*_*e*_ = 1.35 × 10^27^ m^−3^, from which the Fermi wave vector follows, *k*_F_ = 3.42 × 10^9^ m^−1^, and the Fermi energy, *E*_F_ = 1.15 eV. The effective mass is sensitive to details of the fitting, especially at higher wave vectors where the Kane model does not include interaction with the many other bands encountered. We have confidence in values up to and including *E*_F_. Above *E*_F_, ${m}_{{\rm{eff}}}^{-1}$ is taken as a parameter to fit the data. In our experiments the pump has a frequency of 377.4 THz (*ℏω*_pump_ = 1.56 eV), which is somewhat greater than the Fermi energy. The resonance at *ℏω*_0_ = 1 eV lies well below *ℏω*_pump_ and in our experiment plays no direct role in absorption of pump radiation. Photon absorption involves transfer to the electrons of *momentum* from phonons or defects but negligible transfer of *energy*. Hence electrons absorb almost the full photon energy. This process determines the imaginary part of the permittivity which has been measured by ellipsometry^5^. We calculate that of the order of 15% of the pump energy is absorbed by the ITO. Using the Fermi distribution and eqs. (2) and (3) we calculate an average inverse mass. The corresponding shift in plasma frequency is shown in Fig. 5.

### Extracting plasma frequency dynamics from experiment

#### The two-temperature model

**Fig. 6:**
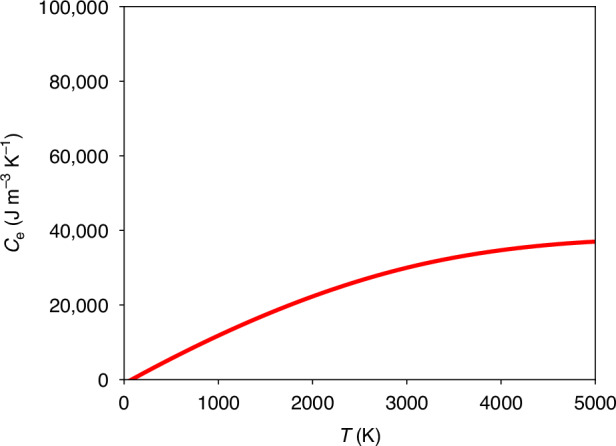
Temperature dependent electron heat capacity, taken from Ref. ^35^. The heat capacity is linear for low electron temperatures, saturating above around 5000 K

The two-temperature model^22^ assumes the electrons and phonons in a material have separate temperatures, *T*_*e*_ and *T*_*l*_, and heat capacities *C*_*e*_ and *C*_*l*_. Assuming the optical pump pulse is partially absorbed, it acts as an energy source, *S*(*t*) that solely heats the electrons. The electrons then equilibrate with the phonons at a rate determined by the electron–phonon coupling constant *g*. Written as a pair of coupled differential equations, this model is,

5 $$\begin{array}{lll}\frac{\partial {T}_{l}}{\partial t} & = & \frac{g}{{C}_{l}}\left({T}_{e}-{T}_{l}\right)\\ \frac{\partial {T}_{e}}{\partial t} & = & \frac{g}{{C}_{e}({T}_{e})}({T}_{l}-{T}_{e})+S(t)\end{array}$$

where the electron–phonon coupling *g* has units of J m^−3^ K^−1^ s^−1^. Here we take the electron heat capacity for ITO from Ref. ^35^ (re–plotted here in Fig. 6) and assume a constant phonon heat capacity of *C*_*l*_ = 2.6 × 10^6^ J m^−3^ K^−1^, as given in^42^. The electron–phonon coupling is temperature dependent^5^, and here we fit it separately to each set of experimental data.

The two equations (5) were integrated numerically using the ‘odeint’ routine in the SciPy library^43^, assuming a Gaussian source with maximum *S*_0_ and full–width–half–maximum $2\Delta \sqrt{ln(2)}$ to represent the heating due to the pump pulse, $S(t)={S}_{0}{[\pi {\Delta }^{2}]}^{-1/2}\exp [-{(t-{t}_{0})}^{2}/{\Delta }^{2}]$. The amplitude of this Gaussian, *S*_0_ and its central time, *t*_0_ are fitting parameters, whereas the FWHM is known and taken as 44 fs. We assume instantaneous equilibrium and insert the electron temperature *T*_*e*_ into the Fermi–Dirac distribution *n*_F_(2) and fix the chemical potential so that the carrier density *ρ*_*e*_ has a set value. The time–variation of the effective mass of the carriers is then computed as follows (see Eq. (3)),

6 $$\frac{1}{\langle {m}_{{\rm{eff}}}(t)\rangle }=-\frac{1}{3{\pi }^{2}{\rho }_{e}\hslash }{\int }_{0}^{\infty }{k}^{2}\,{\rm{d}}k\frac{\partial \omega }{\partial k}\frac{\partial {n}_{{\rm{F}}}}{\partial k}$$

The time–varying plasma frequency *ω*_p_(*t*), is thus, as stated in the main text,

7 $${\omega }_{{\rm{p}}}^{2}(t)=\frac{{\rho }_{e}{e}^{2}}{{\epsilon }_{0}\,\langle {m}_{{\rm{eff}}}(t)\rangle }$$

In our comparison to the experimental data, we take the carrier density *ρ*_*e*_, the electron-phonon coupling *g*, the pulse arrival time *t*_0_, and the delivered pump energy, *S*_0_ as fitting parameters. The fitted values for six of the data sets are given in Table 1. We note that the value of the electron–phonon coupling of around 5 × 10^16^ J K^−1^ m^−3^ is close to the theoretically computed values given in Ref. ^35^. The carrier density is consistent at around 1.2 × 10^27^ m^−3^, in agreement with the reported range^44^, as is the collision rate at 2 × 10^14^ rad s^−1^. Note that the calculated shift in plasma frequency depends on the value of *γ*, the reflectivity changing more rapidly with respect to wavelength when the collision rate is lower. Here we constrained *γ* to the range [2, 2.4] × 10^14^ rad s^−1^. Finally, for a 44 fs pump pulse, we can estimate the total power delivered at the surface to be e.g, 2.27 × 10^16^ × 44 × 10^−15^J m^−2^ = 998 J m^−2^. Taking an upper bound where this energy is uniformly absorbed into the 310 nm ITO layer, the absorbed energy density is 3.22 × 10^9^ J m^−3^. Comparison with the fitted values of *S*_0_ in the table below indicates that the absorbed energy is a few percent of this upper bound.

**Table 1:** Parameter values, fitting a two-temperature model and calculated reflection and transmission to the experimental data

Parameter/Intensity (TW/cm^2^)	2.27	2.75	3.47	3.95	5.98	10.28
*t*_0_ ( × 10^−12^ s)	0.577	0.557	0.588	0.599	0.610	0.571
*S*_0_ ( × 10^7^ J m^−3^)	4.73	6.26	7.07	8.41	7.97	4.38
*g* ( × 10^16^ J K^−1^ m^−3^ s^−1^)	4.376	4.01	3.767	4.63	3.265	1.558
*ρ*_*e*_ ( × 10^27^ m^−3^)	1.19	1.21	1.22	1.22	1.22	1.16
*γ* ( × 10^14^ rad s^−1^)	2	2	2	2	2	2

#### Operator theory of reflection/transmission

Having determined the time variation of the plasma frequency via Eq. (7), we now calculate the reflection and transmission of optical pulses through the layer of modulated ITO, into a glass substrate. For this, we use the operator method described in Ref. ^40^, specifying the properties of the ITO layer through a time–varying conductivity, as also described in^40^. This method is formally very similar to the standard multilayer scattering/transfer matrix approach, differing only in the inclusion of the coupling between different frequencies, due to the time modulation. The reason for using this theory is that, in our fitting to the experiment we must use *both* the frequency shifts and the changes in reflectivity/transmissivity (which we only have values for relative to the unmodulated sample).

Note that in the following, there is a difference of operator ordering compared to our previous work in e.g., Ref. ^30^. This arises from our use of a time–modulated current, ***j***, rather than a time–modulated polarisation, ***P***. These two quantities are no longer simply related by ***j*** = − i*ω****P***, as they are in stationary media, and the choice has an effect on the formulae, although numerically this difference is small.

To establish how the time–varying plasma frequency (7) enters Maxwell’s equations, we start from the following expression for the electrical current density in the material,

8 $${\boldsymbol{j}}(t)=2e\int \frac{{{\rm{d}}}^{3}{\boldsymbol{k}}}{{(2\pi )}^{3}}{n}_{{\rm{F}}}\left({\boldsymbol{k}}-\Delta {\boldsymbol{k}}(t),t\right)\frac{\partial \omega ({\boldsymbol{k}})}{\partial {\boldsymbol{k}}}$$

where, as above, *n*_F_ is the Fermi–Dirac distribution, and the prefactor of two counts two spin states per momentum state. The derivative ∂*ω*/∂***k*** is the electron group velocity, and thus Eq. (8) represents the electron charge times the group velocity summed over the electron distribution in the conduction band. Eq. (8) includes two time dependences in the electron distribution that are somewhat analogous to the ‘fast’ and ‘slow’ nonlinearities identified in Ref. ^39^. The first is due to the electric field acting on the electrons on the timescale of a single optical cycle, which acts to shift the momentum distribution by Δ***p***(*t*) = *ℏ*Δ***k***(*t*). There should also be a spreading of the distribution due to collisions, which results in a non–equilibrium electron distribution, but we do not consider this here. The second time dependence in the Fermi–Dirac distribution is due to the heating of the electrons from the absorbed intensity from the pump and is calculated from the two–temperature model described in Sec. 5.2.1. This second time scale corresponds to many cycles of both pump and probe pulses.

In the following, we neglect the short timescale dynamics of *n*_F_ due to the pump, considering only the ‘slow’ nonlinearity, and taking Δ***k*** to be the small shift in wavevector due to the probe. We thus expand the current to leading order in Δ***k***, giving,

9 $$\begin{array}{lcl}{\boldsymbol{j}}(t) & \sim & 2e\displaystyle\int \displaystyle\frac{{{\rm{d}}}^{3}{\boldsymbol{k}}}{{(2\pi )}^{3}}\left[{n}_{{\rm{F}}}({\boldsymbol{k}},t)-\Delta {\boldsymbol{k}}(t)\cdot \displaystyle\frac{\partial {n}_{{\rm{F}}}({\boldsymbol{k}},t)}{\partial {\boldsymbol{k}}}\right]\displaystyle\frac{\partial \omega ({\boldsymbol{k}})}{\partial {\boldsymbol{k}}}\\ & = & -\displaystyle\frac{2e}{\hslash }\Delta {\boldsymbol{p}}(t)\cdot \displaystyle\int \displaystyle\frac{{{\rm{d}}}^{3}{\boldsymbol{k}}}{{(2\pi )}^{3}}{{\boldsymbol{e}}}_{k}\otimes {{\boldsymbol{e}}}_{k}\displaystyle\frac{\partial {n}_{{\rm{F}}}(k,t)}{\partial k}\displaystyle\frac{\partial \omega (k)}{\partial k}\\ & = & -\displaystyle\frac{e}{3{\pi }^{2}\hslash }\Delta {\boldsymbol{p}}(t){\displaystyle\int }_{0}^{\infty }{k}^{2}\,{\rm{d}}k\displaystyle\frac{\partial {n}_{{\rm{F}}}(k,t)}{\partial k}\displaystyle\frac{\partial \omega (k)}{\partial k}\end{array}$$

where ***e***_*k*_ = ***k***/*k*, and the current in the absence of an applied field was taken to be zero. To find the shift in momentum Δ***p***, we take the electrons to obey the equation of a free particle undergoing inelastic collisions at a rate *γ*, ∂_*t*_***p*** + *γ****p*** = *e****E***, and the net change in momentum is thus,

10 $$\begin{array}{rcl}\Delta {\boldsymbol{p}}(t) & = & e{\displaystyle\int }_{-\infty }^{t}{\rm{d}}{t}^{{\prime} }{{\rm{e}}}^{-\gamma (t-{t}^{{\prime} })}{\boldsymbol{E}}({t}^{{\prime} })\\ & = & \displaystyle\frac{e{{\boldsymbol{E}}}_{0}{{\rm{e}}}^{-{\rm{i}}\omega t}}{-{\rm{i}}\omega +\gamma }\end{array}$$

where in the second line we took the electric field of the probe to be monochromatic, ***E*** = ***E***_0_e^−i*ωt*^. Combining Eqns. (9) and (10) we can thus identify the time–varying conductivity of our ITO layer, *σ*(*ω*, *t*) as equal to

11 $${\boldsymbol{j}}(t)=\frac{{\rho }_{e}{e}^{2}}{\langle {m}_{{\rm{eff}}}(t)\rangle }\frac{{\rm{i}}}{\omega +{\rm{i}}\gamma }{{\boldsymbol{E}}}_{0}{{\rm{e}}}^{-{\rm{i}}\omega t}={\epsilon }_{0}\frac{{\rm{i}}{\omega }_{{\rm{p}}}^{2}(t)}{\omega +{\rm{i}}\gamma }{{\boldsymbol{E}}}_{0}{{\rm{e}}}^{-{\rm{i}}\omega t}=\sigma (\omega ,t){{\boldsymbol{E}}}_{0}{{\rm{e}}}^{-{\rm{i}}\omega t}$$

In the above we have identified 〈*m*_eff_〉 from Eq. (6). For a general probe field, we sum over all frequencies, replacing the constant vector amplitude ***E***_0_ with the Fourier amplitude $\widetilde{{\boldsymbol{E}}}(\omega )$. The current can then be re–written in a form appropriate for the operator methods described in^40^,

12 $$\begin{array}{rcl}{\boldsymbol{j}} & = & {\displaystyle\int }_{-\infty }^{\infty }\displaystyle\frac{{\rm{d}}\omega }{2\pi }\sigma (\omega ,t)\widetilde{{\boldsymbol{E}}}(\omega ){{\rm{e}}}^{-{\rm{i}}\omega t}\\ & = & {\displaystyle\int }_{-\infty }^{\infty }\displaystyle\frac{{\rm{d}}\omega }{2\pi }\widetilde{{\boldsymbol{E}}}(\omega ){\widehat{\sigma }}_{{\rm{R}}}(\omega ,{\rm{i}}{\partial }_{\omega }){{\rm{e}}}^{-{\rm{i}}\omega t}\\ & = & {\displaystyle\int }_{-\infty }^{\infty }\displaystyle\frac{{\rm{d}}\omega }{2\pi }{{\rm{e}}}^{-{\rm{i}}\omega t}{\widehat{\sigma }}_{{\rm{L}}}(\omega ,-{\rm{i}}{\partial }_{\omega })\widetilde{{\boldsymbol{E}}}(\omega )\end{array}$$

In the second line of (12) we have re–written the conductivity as an operator. This is done through replacing the time variable in the conductivity with a derivative i∂_*ω*_ that acts on the exponential $\exp (-{\rm{i}}\omega t)$. Of course, for this to work, we must first take all the time dependence in the expression to the *right* of all the frequency dependence, hence the subscript ‘*R*’. In the final line we integrated by parts to shift the operator onto the Fourier amplitude $\widetilde{{\boldsymbol{E}}}$, which leads to an extra minus sign in front of the derivative ∂_*ω*_ and swaps the operator ordering so now the derivatives in $\widehat{\sigma }$ appear to the *left* of the frequency dependence. In the final line of (12) we can now identify the Fourier components of the electric current density as $\widetilde{{\boldsymbol{j}}}={\widehat{\sigma }}_{L}(\omega ,-{\rm{i}}{\partial }_{\omega }){\boldsymbol{E}}(\omega )$. Maxwell’s equations within the ITO layer can thus be written as,

13 $$\begin{array}{lll}{\boldsymbol{\nabla}}\widetilde{H}\times{{\boldsymbol{e}}}_{z} &=&-{\rm{i}}\widehat{\omega}{\epsilon}_{0}\left[{\epsilon}_{\infty}+{\rm{i}}{\epsilon}_{0}^{-1}{\widehat{\omega}}^{-1}{\widehat{\sigma }}_{{\rm{L}}}\right]\widetilde{{\boldsymbol{E}}}=-{\rm{i}}\widehat{\omega}{\epsilon}_{0}\widehat{\epsilon }\widetilde{{\boldsymbol{E}}}\\ {\boldsymbol{\nabla }}\times \widetilde{{\boldsymbol{E}}}&=& {\rm{i}}\widehat{\omega }{\mu }_{0}\widetilde{H}{{\boldsymbol{e}}}_{z}\end{array}$$

where *ϵ*_*∞*_ is the permittivity at infinite frequency and where we assumed a transverse magnetic (TM) probe, ***H*** = *H****e***_*z*_, propagating in the *x*–*y* plane, and unlike the notation used in Refs. ^30,40^ we have written the frequency as an operator $\widehat{\omega }$, to remind us that in any computation this is a diagonal matrix containing the values of frequency. Combining both of (13) we obtain an operator version of the Helmholtz equation for the out-of-plane magnetic field,

14 $${{\boldsymbol{\nabla }}}^{2}\widetilde{H}+\frac{\widehat{\omega }\,\widehat{\epsilon }\,\widehat{\omega }}{{c}^{2}}\widetilde{H}=0$$

In the experiment, the probe pulse is incident at an angle, *θ* onto the ITO layer. To account for this in a simple way, we take a fixed transverse wavevector ${k}_{y}=\frac{{\omega }_{0}}{c}\sin (\theta )$ where *ω*_0_ is the central frequency of the impinging probe, and thus from (14) we can identify the wavevector operator, ${\widehat{K}}^{2}=\frac{1}{{c}^{2}}\omega \widehat{\epsilon }(\omega ,-{\rm{i}}{\partial }_{\omega })\omega -{k}_{y}^{2}\,\widehat{1}$. Just as for the ordinary scalar Helmholtz equation, Eq. (14) has the formal solutions $\widetilde{H}(x,\omega )=\exp (\pm {\rm{i}}\widehat{K}x)\left|{\widetilde{H}}_{0}(\omega )\right\rangle$, where $\left|{\widetilde{H}}_{0}\right\rangle$ is the Fourier spectrum of the magnetic field at *x* = 0, which we write as a vector, indicating that this is treated as a vector in numerical calculation.

To solve the reflection problem for waves incident onto the ITO layer plus glass substrate, we now solve the general problem of incidence onto an interface between two materials where the wavevector operators are ${\widehat{K}}_{1}$ and ${\widehat{K}}_{2}$ and the permittivity operators are ${\widehat{\epsilon }}_{1}$ and ${\widehat{\epsilon }}_{2}$ (as is typically the case at optical frequencies, we take the magnetic permeability as unity everywhere). Taking the interface at *x* = 0 and writing the field in medium 1 as $\left|{\widetilde{H}}_{1}(x)\right\rangle =\exp ({\rm{i}}{\widehat{K}}_{1}x)\left|{\widetilde{H}}_{{\rm{in}}}\right\rangle +{\widehat{R}}_{12}\exp (-{\rm{i}}{\widehat{K}}_{1}x)\left|{\widetilde{H}}_{{\rm{in}}}\right\rangle$, and in medium 2 as $\left|{\widetilde{H}}_{2}(x)\right\rangle ={\widehat{T}}_{12}\exp (-{\rm{i}}{\widehat{K}}_{2}x)\left|{\widetilde{H}}_{{\rm{in}}}\right\rangle$, the continuity of the magnetic field across the interface leads to

15 $$\widehat{1}+{\widehat{R}}_{12}={\widehat{T}}_{12}$$

and

16 $$\widehat{1}-{\widehat{R}}_{12}={\widehat{K}}_{1}^{-1}\widehat{\omega }{\widehat{\epsilon }}_{1}{\widehat{\epsilon }}_{2}^{-1}{\widehat{\omega }}^{-1}{\widehat{K}}_{2}{\widehat{T}}_{12}={\widehat{Z}}_{12}{\widehat{T}}_{12}$$

Combining the two operator eqs. (15) and (16) we can then find expressions for the reflection and transmission operators

17 $$\begin{array}{rcl}{\widehat{T}}_{12} & = & 2{(\widehat{1}+{\widehat{Z}}_{12})}^{-1}\\ {\widehat{R}}_{12} & = & (\widehat{1}-{\widehat{Z}}_{12}){(\widehat{1}+{\widehat{Z}}_{12})}^{-1}\end{array}$$

which are simply operator analogues of the usual interface reflection and transmission coefficients. With the results (17) we can give the reflection and transmission operators for a system composed of vacuum (medium 1, ${\widehat{\epsilon }}_{1}=\widehat{1}$), an ITO layer of thickness *d* (medium 2, *d* = 310 nm in the experiment), and a glass substrate (medium 3, ${\widehat{\epsilon }}_{3}=2.25\,\widehat{1}$). We can construct the operators for the multilayer through adding in the operators for all the possible reflections. The reflection operator for the complete system is thus,

18 $$\begin{array}{rcl}\widehat{R} & = & {\widehat{R}}_{12}+{\widehat{T}}_{21}{{\rm{e}}}^{{\rm{i}}{\widehat{K}}_{2}d}{\widehat{R}}_{23}{{\rm{e}}}^{{\rm{i}}{\widehat{K}}_{2}d}{\widehat{T}}_{12}+{\widehat{T}}_{21}{{\rm{e}}}^{{\rm{i}}{\widehat{K}}_{2}d}{\widehat{R}}_{23}{{\rm{e}}}^{{\rm{i}}{\widehat{K}}_{2}d}{\widehat{R}}_{21}{{\rm{e}}}^{{\rm{i}}{\widehat{K}}_{2}d}{\widehat{R}}_{23}{{\rm{e}}}^{{\rm{i}}{\widehat{K}}_{2}d}{\widehat{T}}_{12}+\ldots \\ & = & {\widehat{R}}_{12}+{\widehat{T}}_{21}{{\rm{e}}}^{{\rm{i}}{\widehat{K}}_{2}d}{\widehat{R}}_{23}{{\rm{e}}}^{{\rm{i}}{\widehat{K}}_{2}d}{\left[\widehat{1}-{\widehat{R}}_{21}{{\rm{e}}}^{{\rm{i}}{\widehat{K}}_{2}d}{\widehat{R}}_{23}{{\rm{e}}}^{{\rm{i}}{\widehat{K}}_{2}d}\right]}^{-1}{\widehat{T}}_{12}\end{array}$$

Similarly, the transmission operator is

19 $$\begin{array}{rcl}\widehat{T} & = & {\widehat{T}}_{23}{{\rm{e}}}^{{\rm{i}}{\widehat{K}}_{2}d}{\widehat{T}}_{12}+{\widehat{T}}_{23}{{\rm{e}}}^{{\rm{i}}{\widehat{K}}_{2}d}{\widehat{R}}_{21}{{\rm{e}}}^{{\rm{i}}{\widehat{K}}_{2}d}{\widehat{R}}_{23}{{\rm{e}}}^{{\rm{i}}{\widehat{K}}_{2}d}{\widehat{T}}_{12}+\ldots \\ & = & {\widehat{T}}_{23}{{\rm{e}}}^{{\rm{i}}{\widehat{K}}_{2}d}{\left[\widehat{1}-{\widehat{R}}_{21}{{\rm{e}}}^{{\rm{i}}{\widehat{K}}_{2}d}{\widehat{R}}_{23}{{\rm{e}}}^{{\rm{i}}{\widehat{K}}_{2}d}\right]}^{-1}{\widehat{T}}_{12}\end{array}$$

To compare to experimental data, we use Eqns. (18) and (19) to compute the above operators, $\widehat{R}$ and $\widehat{T}$. All operators are evaluated as matrices, and functions of matrices (using the Python SciPy library^43^), with the frequency derivatives appearing in Eq. (12) written using matrices enacting a Fourier transform $\widehat{{\mathcal{F}}}$, i.e. ${\partial }_{\omega }\equiv {\rm{i}}\widehat{{\mathcal{F}}}\widehat{t}{\widehat{{\mathcal{F}}}}^{-1}$, with $\widehat{t}$ a diagonal matrix containing all the time values in the simulation. The reflected power is computed as ${P}_{{\rm{R}}}={\eta }_{1}\left\langle {\widetilde{H}}_{{\rm{in}}}\right|{\widehat{R}}^{\dagger }\widehat{R}\left|{\widetilde{H}}_{{\rm{in}}}\right\rangle$, where *η*_1_ is the impedance of vacuum. Similarly the transmitted power is ${P}_{{\rm{T}}}={\eta }_{3}\left\langle {\widetilde{H}}_{{\rm{in}}}\right|{\widehat{T}}^{\dagger }\widehat{T}\left|{\widetilde{H}}_{{\rm{in}}}\right\rangle$, where ${\eta }_{3}={\eta }_{1}/\sqrt{{\epsilon }_{3}}$ is the impedance of the glass substrate. To calculate the average frequency of the reflected/transmitted waves, we compute $\left\langle {H}_{{\rm{in}}}\right|{\widehat{R}}^{\dagger }\widehat{\omega }\widehat{R}\left|{\widetilde{H}}_{{\rm{in}}}\right\rangle /\left\langle {\widetilde{H}}_{{\rm{in}}}\right|{\widehat{R}}^{\dagger }\widehat{R}\left|{\widetilde{H}}_{{\rm{in}}}\right\rangle$ and $\left\langle {H}_{{\rm{in}}}\right|{\widehat{T}}^{\dagger }\widehat{\omega }\widehat{T}\left|{\widetilde{H}}_{{\rm{in}}}\right\rangle /\left\langle {\widetilde{H}}_{{\rm{in}}}\right|{\widehat{T}}^{\dagger }\widehat{T}\left|{\widetilde{H}}_{{\rm{in}}}\right\rangle$. These values are converted to wavelengths to compare with experimental data.

## Supplementary information

Supplementary Information

## Data Availability

The data will be made available upon reasonable request to the authors.

## Code availability

The code will be made available upon reasonable request to the authors.
