# Supplementary material for: Ultrafast switching of optical properties in a doped semiconductor by intense femtosecond laser pulses
Source: Light Sci Appl. 2026 Aug 4;15:336. doi: 10.1038/s41377-026-02346-x (PMC13434625; doi:10.1038/s41377-026-02346-x)
Supplement: Supplementary file 1 [file 41377_2026_2346_MOESM1_ESM.pdf]

Supplementary Information

Yan Li[1†], Stefano Vezzoli[1†], Tim Klee[1], Joseph J Broughton[1], Romain Tirole[1], Anthony C Harwood[1], Hortense Allegre[1], Simon A R Horsley2[†], Riccardo Sapienza[1], John B Pendry1[*†], John W G Tisch[1]

[1]*The Blackett Laboratory, Department of Physics, Imperial College London, Exhibition Rd, London, SW7 2BW, United Kingdom.

[2]Department of Physics and Astronomy, Centre for Metamaterial Research and Innovation, University of Exeter, Stocker Road, Exeter, EX4 4QL, United Kingdom.

1. Sample fabrication

The sample is a commercially available 310 nm ITO coating on a 1.1 mm float glass from Präzisions Glas & Optik GmbH.

2. Profiles of the pump and probe pulses

Pump and probe pulses are derived from a Ti:Sapphire chirped pulse amplification laser system (Femtopower, Femtolasers) operating at a wavelength of approximately 795 nm. The probe pulses at 1240 nm are produced from a portion of the 795 nm pulses sent into an optical parametric amplifier (TOPAS, Light Conversion) with approximately 50 fs temporal FWHM. The temporal profiles are characterized by a home-built second-harmonic-generation frequency-resolved optical gating (SHG-FROG) device. All pulses are approximately Gaussian in their temporal profile. The spatial profiles are characterized at a plane equivalent to the focus at the ITO sample using a beam profiler (Thorlabs). Spectra are acquired using a commercial spectrometer (NIRQuest, Ocean Optics). Delay scans are performed using a computer-controlled delay stage (Zaber Technologies).

3. Measurement of the ITO pump-probe delay scan

We use custom software to control the delay scan. The software interface communicates with both the spectrometer and the delay stage. The starting pump-probe delay is set ~600 fs before the pump intersects the probe, with a full scan range of 3 ps. At each delay step of 5 fs, the probe spectrum is recorded. From the raw data, we extract the spectral region of interest between 1100 nm and 1400 nm, where background contributions are negligible. This selected 300 nm range significantly exceeds the FWHM of the probe spectrum. We integrate the spectra over the wavelength range to obtain the total spectral intensity versus delay for all pump intensities. The central wavelength of the probe is calculated using a weighted average, and wavelength shifts are obtained by comparing with the mean wavelength of the probe when no pump is present. We

calibrate the zero delay (t_0 = 0 fs) as the time when the net wavelength shift is zero in the transmitted light.

4. Float glass substrate check

Since the sample consists of a 310 nm thin film on a 1.1 mm float glass substrate and is pumped at high intensity, additional nonlinear effects such as cross-phase modulation (XPM) inevitably occur during propagation through the substrate, modulating the probe spectrum. To isolate these contributions, we perform identical pump–probe scans on a bare float glass substrate of the same thickness and compare the results with those from the ITO sample at similar pump intensities.

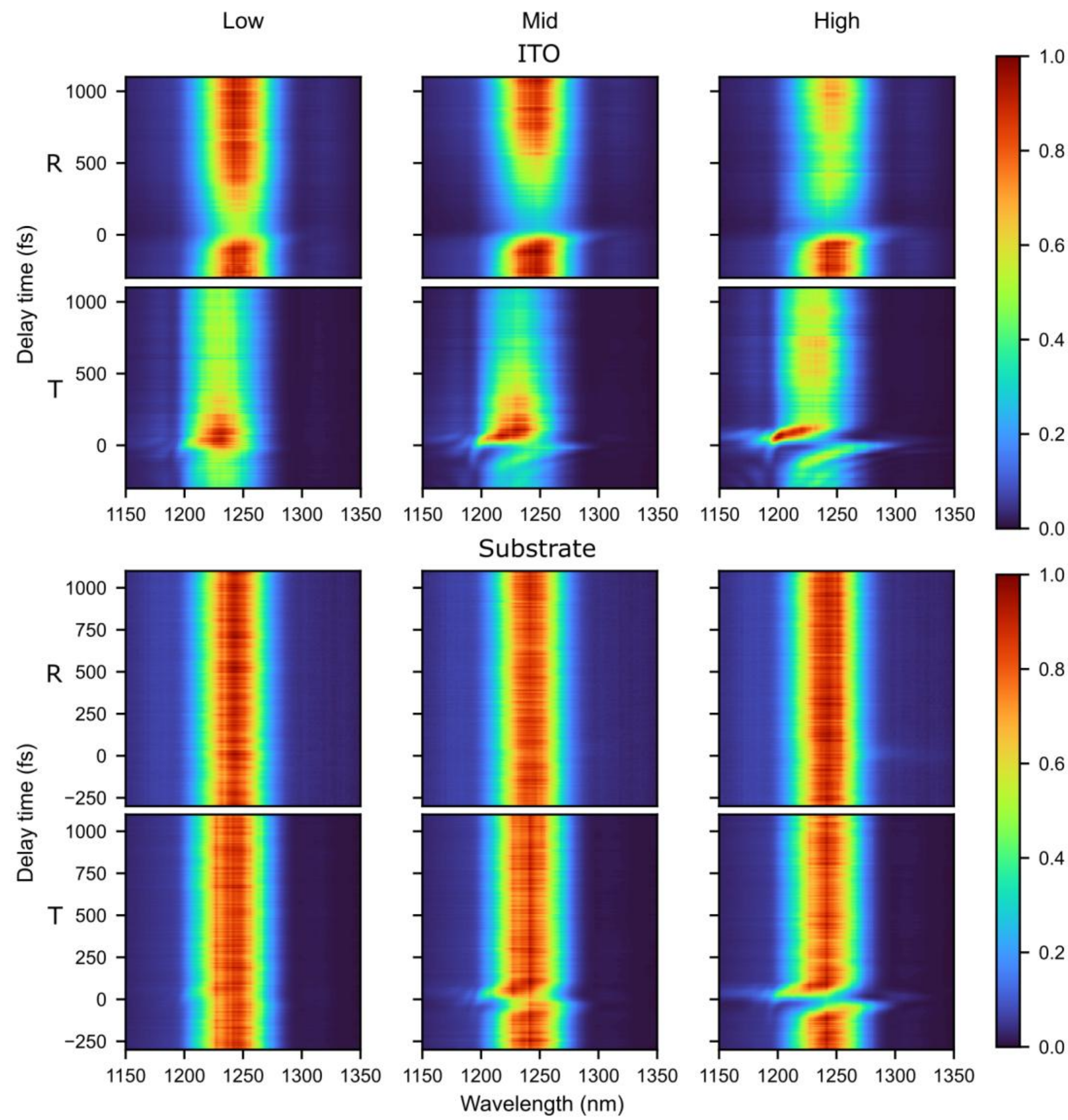

Fig. A1: Pump-probe scan performed on the float glass substrate in low-, medium-, and high-intensity regimes, respectively.

As shown in Fig. A1 and Fig. A2, the float glass substrate exhibits a clearly distinct response. First, the float glass shows no pump-induced modulation of reflectivity or transmissivity, except at high pump intensities where a reduction in transmissivity is observed, opposite to the ITO response, which exhibits an increase. This will lead to partial cancellation of the overall transmissivity modulation. Nonetheless, the relaxation behaviour of the glass shows clear discrepancy to ITO's. Second, no redshift is observed in the reflected probe at low to medium pump intensities. At the highest intensities, the float glass does contribute to a small redshift in reflection, but no blueshift is seen. This suggests that the blueshift is a distinctive high-intensity response of ITO, which we suggest is linked to Auger processes. In contrast, nonlinear spectral shifts in transmission begin to emerge at low to medium intensities, attributed to XPM. Since the pump intensity is extremely high, we caution that the transmitted probe spectrum alone is insufficient to fully characterise the ITO response.

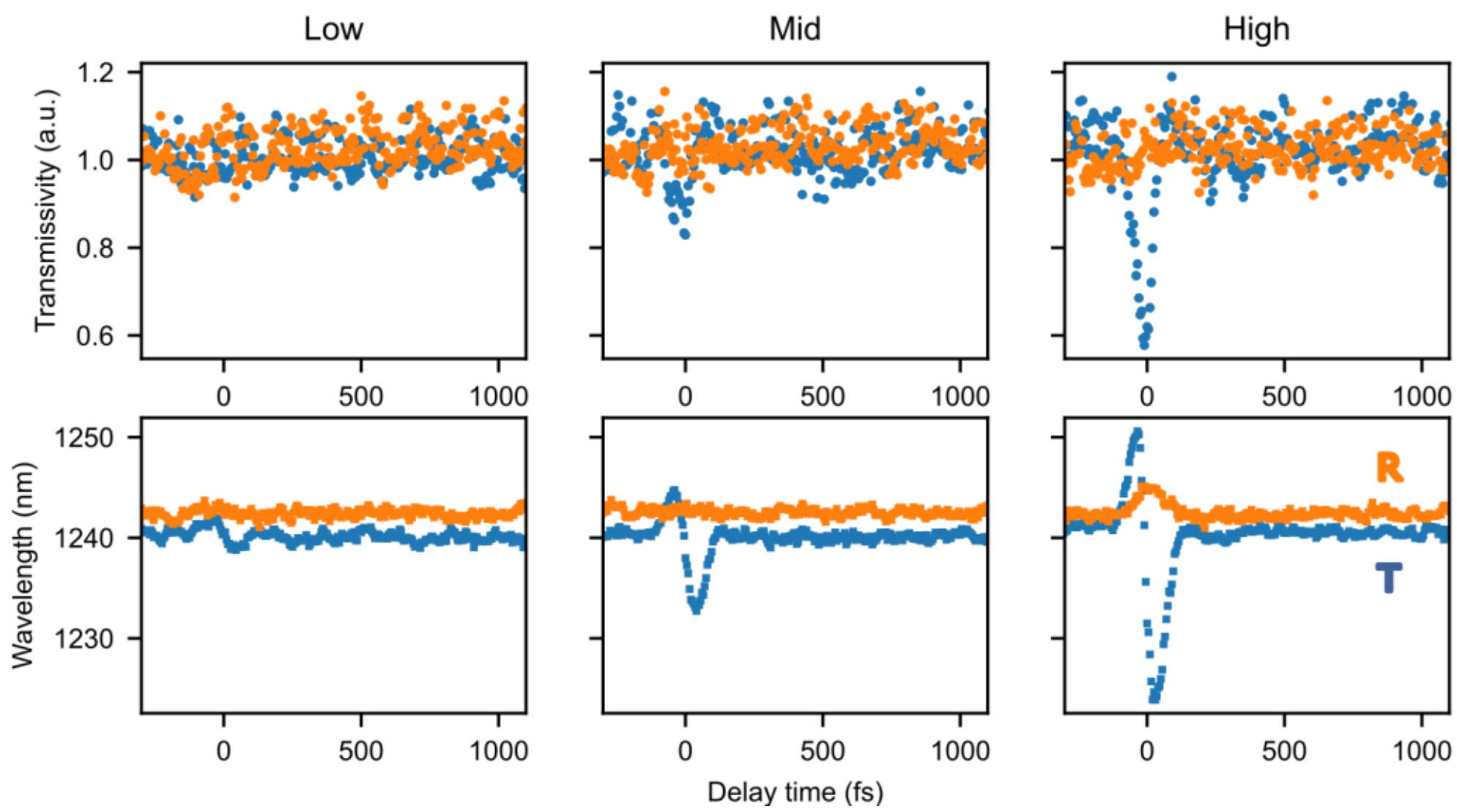

Fig. A2: Integrated spectrum and wavelength shift of the transmitted probe from the substrate in low-, medium-, and high-intensity regimes, respectively.

5. Ellipsometry & complex permittivity data

We have conducted ellipsometry of the 310 nm ITO sample and fitted the parameters to the Drude model. The extracted real and imaginary components of the complex permittivity are shown in Fig. A3.

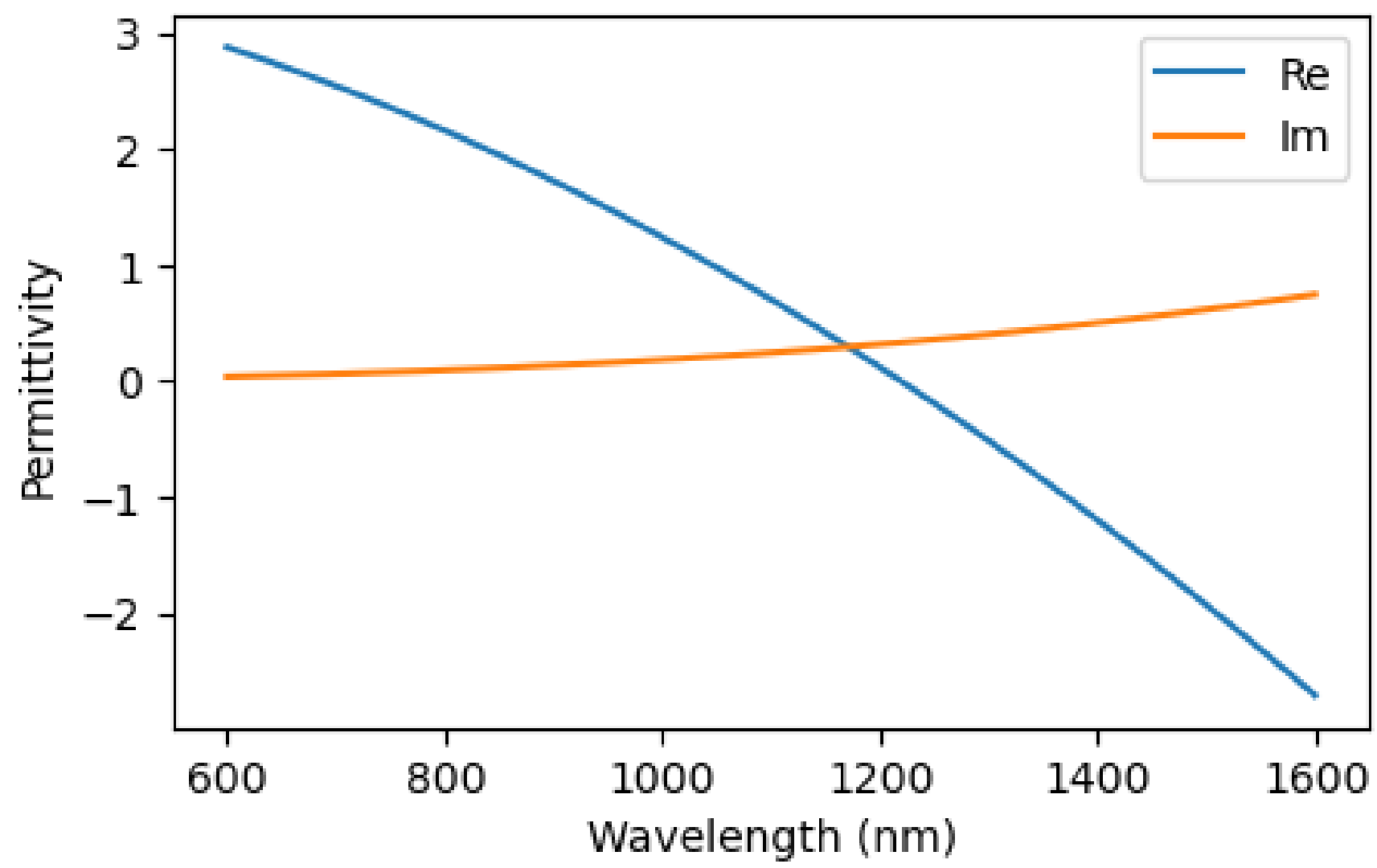

Fig. A3: The real and imaginary permittivity of the 310 nm ITO sample extracted from the ellipsometry measurement.

5
